# Late Steps in Secretory Lysosome Exocytosis in Cytotoxic Lymphocytes

**DOI:** 10.3389/fimmu.2013.00359

**Published:** 2013-11-18

**Authors:** Peter van der Sluijs, Mallik Zibouche, Peter van Kerkhof

**Affiliations:** ^1^Department of Cell Biology, University Medical Center Utrecht, Utrecht, Netherlands

**Keywords:** lytic granules, secretory lysosomes, maturation, endosomes, degranulation

## Abstract

Natural Killer cells are a subset of cytotoxic lymphocytes that are important in host defense against infections and transformed cells. They exert this function through recognition of target cells by cell surface receptors, which triggers a signaling program that results in a re-orientation of the microtubule organizing center and secretory lysosomes toward the target cell. Upon movement of secretory lysosomes to the plasma membrane and subsequent fusion, toxic proteins are released by secretory lysosomes in the immunological synapse which then enter and kill the target cell. In this minireview we highlight recent progress in our knowledge of late steps in this specialized secretion pathway and address important open questions.

Natural Killer (NK) cells and cytotoxic T lymphocytes (CTL) are essential effectors of innate and adaptive immunity against infected or nascent cancer cells (1). The cytotoxic process is initiated by recognition of target cells via immune receptors and accessory plasma membrane proteins (2). CTL are activated via the T cell receptor (TCR) that interacts with MHC class I molecules and cognate antigenic peptides derived from target cells. NK cytotoxicity occurs via ligation of activating receptors and is kept in check by recognition of self MHC class I molecules. Cytotoxic lymphocytes use two non-redundant pathways to execute their effector functions. The first of which involves the interaction of Fas ligand on CTL and NK cells with Fas receptor on the target cell, which causes receptor oligomerization and apoptosis of the target cell (3). The second pathway also known as the lytic granule pathway represents a rapid and efficient exocytic transport route of vesicular structures containing the lytic molecules perforin and granzymes and is the focus of this minireview.

The lytic granule pathway involves the vectorial and polarized trafficking of the granules toward the immunological synapse and the re-organization of the microtubule organizing center toward the target cell and subsequent release of cytotoxic proteins. Besides the cytotoxic proteins, the granules also contain lysosomal enzymes, an acidic proteoglycan core, and lysosome-associated membrane proteins. Since this content is released by outside in signaling, lytic granules represent a hybrid organelle with shared properties of lysosomes and secretory granules and are therefore also called secretory lysosomes or lysosome related organelles (4, 5).

An essential driver for the development of our understanding of the lytic pathway was the presentation of patients with Familial Hemophagocytic Lymphohistiocytosis (FHL), whose cytotoxic lymphocytes fail to kill target cells. As a consequence of which patients suffer from uncontrolled and massive lymphocyte and macrophage proliferation. A group of genes has been discovered in which mutations causes strongly impaired lytic capacity. These include *RAB27A* (Griscelli syndrome type 2, GS2), *UNC13D* (FHL3), *STX11* (FHL4), and *STX-BP2* (FHL5), which encode proteins of conserved families that regulate membrane trafficking (4). It is thought that the related disease phenotype of the cytotoxic lymphocytes, reflects the functional organization of the wild type forms of these proteins in a network that cooperatively regulates the degranulation pathway. Although this is a widely held belief, our understanding of the molecular mechanisms coupling their function in regulating the degranulation pathway and how they are controlled by upstream signaling is limited.

Even though NK cells and CTL functions belong to distinct arms of immunity, mutations in genes causing FHL produce dramatic yet remarkably related cytoarchitectural and immunodeficiency phenotypes. In addition, primary CTL and NK cell responses reveal similar molecular requirements (6, 7). Collectively this supports the notion of a common principle for granule exocytosis in cytotoxic lymphocytes on which cell-type specific layers of signaling are superimposed. We therefore will not distinguish between the lytic granule pathway in CTL and NK in our discussion.

## Maturation of Secretory Lysosomes

**Figure 1:**
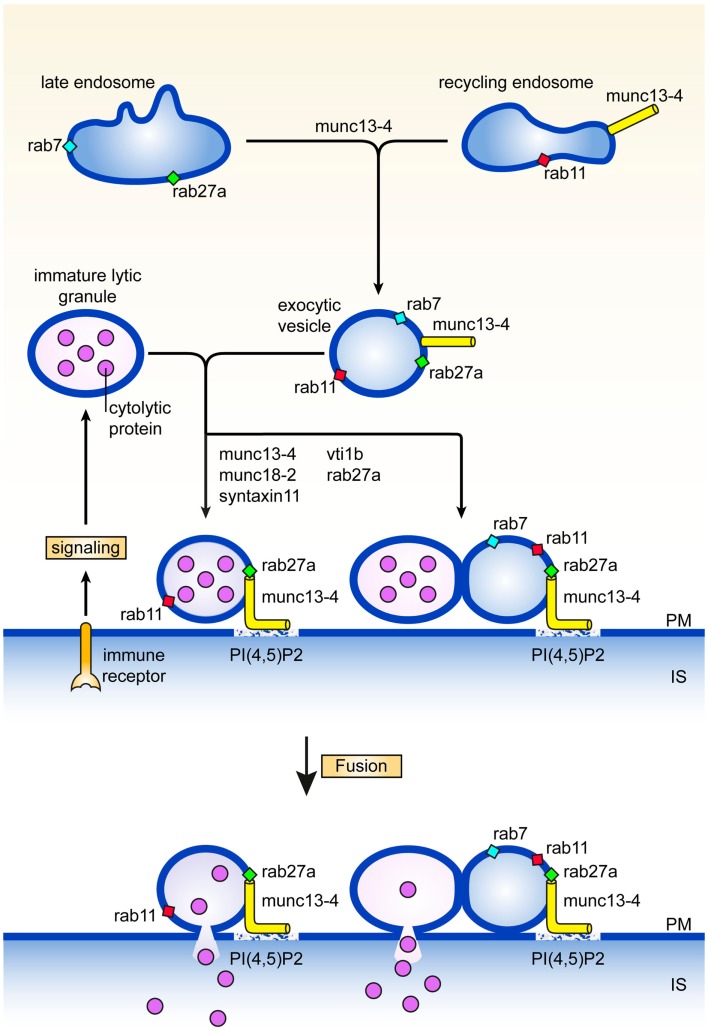
**Maturation and fusion**. Pathways and proteins regulating various aspects of the maturation and fusion of secretory lysosomes (see text for details). PM denotes plasma membrane, IS immunological synapse.

Careful electronmicroscopy studies in CTL, mast cells, and melanocytes revealed a heterogenous appearance of secretory lysosomes in terms of cytoplasmic localization, size, and electron dense luminal matrix (8–10). Kinetic tracer uptake experiments and antibody labeling on ultrathin sections suggested the presence of three or more classes of secretory lysosomes, that likely represent different stages in the formation of mature secretory lysosomes. Secretory lysosomes are formed from precursor organelles through a series of distinct membrane transport steps that continuously deliver house-keeping content and specific effector molecules from endosomes, the trans Golgi network, and the cytoplasm (11). How the pathways between them and with other granules are regulated is only recently being explored (12–15). An early stage in the formation of secretory lysosomes involves the merger of recycling endosomes containing rab11 and munc13-4 with late endosomes characterized by the presence of rab27a and rab7, to a so called exocytic endosome (Figure 1). The coalescence of the two endocytic structures might serve to bring exocytic traffic regulatory proteins that are needed at later stages of the secretory lysosome release. Since rab11 does not bind munc13-4 (13), other small GTPases could provide a link between munc13-4 and the endosomal system. Rab15 an endosome-localized rab (16) might take on this task as it binds to munc13-4 and coregulates with rab27 the exocytosis of von Willebrandt factor from Weibel–Palade bodies (17), the secretory lysosomes of endothelial cells. The tetrameric adaptor complex AP-1 and kinesin KIF13A represent two other membrane traffic regulators through recycling endosomes that have been implicated in the function of secretory lysosomes (18). A complex of AP-1 and KIF13A is thought to define a recycling endosomal domain partially overlapping with rab11 (18) that could facilitate cargo sorting. The AP-1*KIF13A complex positions endosomes close to secretory lysosomes in the cell periphery thereby facilitating interorganellar connections.

## Tethering Secretory Lysosomes at the Plasma Membrane

A second step in secretory lysosome maturation occurs when the cytotoxic lymphocyte engages itself in an interaction with a target cell during which the exocytic endosomes and associated exocytic machinery move to the immunological synapse in parallel with organelles containing LAMPs, perforin, and granzymes. At least two scenarios are compatible with the available data for the next stage in the secretory lysosome maturation pathway. The two organelles may fuse with each other to generate a structure with lytic and exocytic potential at the immunological synapse. Alternatively, tethering of the exocytic endosome equipped with munc13-4, rab27, and rab11, to the lytic precursor organelle with perforin and granzyme (without actual fusion) is already sufficient to assemble and concentrate the protein machinery for efficient docking and fusion at the plasma membrane (Figure 1). Enhanced tethering of secretory lysosomes that are paired with TCR-containing endosomes has been observed at the immunological synapse of CTL. In effect tethering prolongs the dwelling time of the organelle at the immunological synapse which facilitates the subsequent fusion step with the plasma membrane, and release of lytic content (19). In support of this notion, we uncovered a second role for munc13-4 in tethering secretory lysosomes at the cell surface that is regulated independently from the upstream function of munc13-4 on recycling endosomes. Total Internal Reflection Fluorescence microscopy of cells expressing munc13-4 point mutants defective in rab27a-binding revealed that secretory lysosomes are severely impaired in the characteristic stalling behavior seen upon initiation of immune receptor signaling, showing that a complex between munc13-4 and rab27a is required for the tethering role of munc13-4 at the plasma membrane (12). In neutrophils, munc13-4 was also found to limit mobilization of rab27a positive granules after lipopolysaccharide stimulation and to restrict them at the plasma membrane (20), suggesting a more general role of the rab27*munc13-4 complex in immune cells.

**Figure 2:**
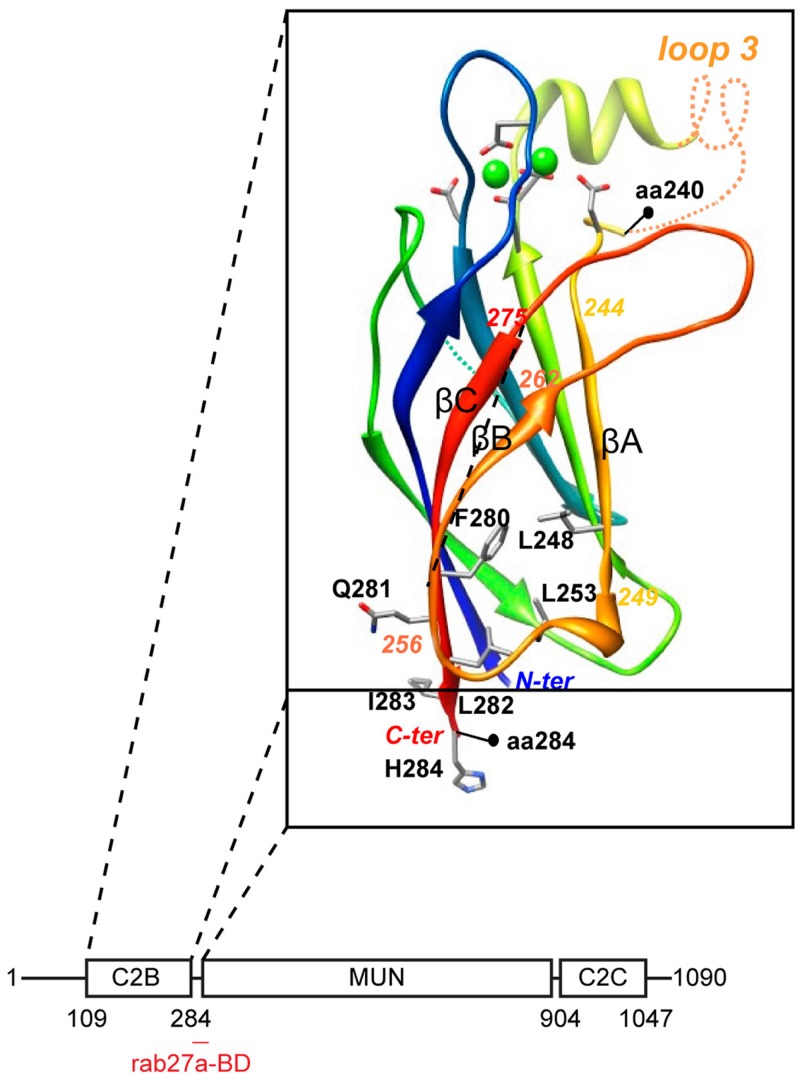
**Domain architecture of munc13-4**. The C2B domain was modeled on the munc13-2 C2B domain structure (26). Note the rab27-binding domain at the junction between C2B and MUN domain.

Molecular insight into the question as to how munc13-4 could serve as a tethering molecule derived from computational structure predictions which revealed very weak homology between the MUN domain and subunits of intracellular membrane tethering complexes (21). The MUN domain is the central region of munc13-4 that is interspaced between the C2B and C2C domains (Figure 2). It contains two conserved munc homology domain sequences (22) that constitute an autonomously folding MUN domain. The MUN domain is conserved in all munc13’s and related proteins and is principally involved in the priming function. The subsequent structure of the MUN domain (23) revealed that it folds into a two-stacked helical bundle, characteristic for the exocyst subunit sec6 and other members of the CATCHR family (complex associated with tethering containing helical rods) of tethering factors. Additional subunits of the oligomeric exocyst, GARP, COG, and Dsl1 tethering complex complexes (24) also fold in this structure as do the cargo binding sites of yeast type V myosins (25). Another common denominator in these complexes is their propensity to bind traffic regulators like rho and rab GTPases, SNAREs, and phospholipids.

We recently characterized the requirements for rab27-binding to munc13-4, and mapped the rab27-binding site to a short sequence just at the junction of the MUN and C2B domains (12) (Figure 2). Since the munc13 C2B domain engages in Ca^2+^- dependent PI(4,5)P2 binding and the C2C domain might constitutively bind membranes, munc13-4 could bridge two membranes via this multiplicity of interactions. CTL and NK cells express two other rab27a effectors, slp1 and slp2-a. Both are non-essential for secretory lysosome release, but they could assist in tethering since their C2 domains are important for membrane binding and they focus tightly at the immunological synapse (27, 28). Since the MUN domain of neuronal munc13 interacts with syntaxin-1 (26), we anticipate that munc13-4 will also bind to a (yet to be identified) syntaxin. In that vein munc13-4 mediated tethering could control specificity in SNARE reactions needed for secretory lysosome fusion.

In contrast to molecules on the secretory lysosome that facilitate tethering and docking at the immunological synapse, the molecular cue(s) on the partnering inner leaflet of the plasma membrane have not yet been defined. Given the analogies between cytokinesis, polarized exocytosis, and degranulation (29) we speculate that exocyst complex subunits could play a role in this pathway. Several lines of evidence are in accordance with this notion. The exocyst subunit sec15 binds directly to rab27 (30), while the capture of secretory granules depends on the rab27a effector MyRIP that interacts directly with exocyst subunits sec6 and sec8 (31, 32). Finally, munc13-4 localizes via its C2B domain to PI(4,5)P2-enriched regions on the plasma membrane (33), while PI(4,5)P2 is also key to localizing exocyst subunits sec3 (34) and exo70 (35). Thus molecules important for polarized secretion in other cell types, are implicated in the degranulation pathway through putative interactions with rab27 and munc13-4.

## Fusion of Secretory Lysosomes with the Plasma Membrane

Tethering of munc13-4, rab27, and rab11 structures with LAMP containing organelles can be considered as a pre-requisite for late endosome-lysosome fusion. In CTL and macrophages this heterotypic fusion event has been shown to involve the Qb SNARE vti1b, which is required for release of secretory lysosome content (19, 36, 37) (Figure 1). Interestingly, vti1b interacts with the Qa SNARE syntaxin-11 in macrophages, where silencing of syntaxin-11 causes formation of enlarged late endosomes and inhibition of late endosome-lysosome fusion. In accordance with this function, syntaxin-11 associates predominantly with compartments of the late endosome-lysosome system in a variety of immune cells. In NK cells it colocalizes with CD-MPR in late endosomes, relocates to the immunological synapse, but is not found on lytic granules (38). In CTL syntaxin-11 localizes to an organelle close to but not on lytic granules (39), while it is found mainly in LAMP-1 and LBPA containing late endosomes in macrophages (37). Loss of syntaxin-11 function causes FHL4 in man (40, 41) and hemophagocytosis in mice (42–44). NK cells and CTL of FHL4 patients normally polarize their secretory lysosomes to the immunological synapse, but fail to release their content. This phenotype is very similar to munc13-4 deficiency in FHL3 patients and strongly argues that syntaxin-11 acts in the same pathway as munc13-4. Unlike for Griscelli type 2 and FHL3 patients, ultrastructural data are not yet available for cytotoxic lymphocytes of FHL4 patients or the recently established syntaxin-11 knock-out mice (42–44). Information on such features as size, position, content, number, etc., of endosomal structures and secretory lysosomes will be required for the evaluation at what stage the lytic pathway is blocked and where syntaxin-11 function is localized with respect of rab27a and munc13-4 activities.

In hemopoietic cells, syntaxin-11 interacts munc18-2 (45, 46), a member of the small family of SM proteins that regulate the activity of SNARE proteins for membrane fusion in time and in space (47). The localization of munc18-2 has been addressed in mast cells where it is associated with late endocytic organelles, just beneath the plasma membrane (48). Mutations in munc18-2 cause FHL5 and also reduce the amount of its partner syntaxin-11, suggesting that munc18-2 acts as a chaperone that stabilizes syntaxin-11 (45, 46). Initially, inactivating munc18-2 mutations appeared to affect degranulation in CTL, NK cells, and neutrophils (49). More recent observations however show that patient mutations of munc18-2 do not only affect cytotoxic lymphocytes but also cause changes in the intestinal and renal epithelium resulting in severe diarrhea and renal proximal tubular dysfunction (50). The fact that additional tissues are affected in FHL5, likely reflects the more ubiquitous expression of munc18-2 compared to munc13-4, and syntaxin-11. Many of the proteins that are described above, act upstream of the final fusion event of secretory lysosomes with the plasma membrane. The implication is that the secretory lysosome pathway might interface with more general fusogenic machinery at or close to the final step. Recent observations of Rettig’s lab support this idea and suggest that munc13-1 (51) and synaptobrevin 2 (52), two mediators of neuronal secretion, act at the final stage of secretory lysosome exocytosis. The daunting challenge now is to piece together which of the SNAREs and associated proteins cooperate in the final stage to fuse the secretory lysosomes with the plasma membrane.

A breakthrough in our understanding of munc13 and munc18 function came from *in vitro* reconstitution assays. Rizo’s lab recently developed an *in vitro* liposome fusion assay in which they could show for the first time a dependency of fusion on munc18 and munc13 (53). Although this was accomplished in combination with SNAREs involved in synaptic vesicle fusion, it will likely provide a general explanation for the role of munc13 and munc18 proteins in membrane fusion. Ma et al. discovered that the closed form of syntaxin is clamped in a tight complex with munc18 and cannot bind other SNAREs. Munc13 can extract syntaxin through a interaction of the MUN domain with syntaxin and possibly munc18, thereby catalyzing the formation of the full syntaxin-SNAP-25-synaptobrevin complex and fusion (26, 53).

## Coupling Fusion of Secretory Lysosomes with Endocytic Retrieval

The trafficking proteins that regulate degranulation of secretory lysosomes are long lived. After exocytic release of content, these are in principle available for re-utiliatization in a next encounter with a target cell. A seminal paper from Eric Long’s lab showed that lytic granules of NK cells undergo both complete and incomplete fusion with the plasma membrane, and suggests that incomplete fusion may promote efficient recycling of lytic granule membrane and proteins (54). SNAREs involved in fusion with the plasma membrane are returned by retrograde endocytic transport (55, 56).

It is less well understood how cytoplasmic proteins like rab27 and munc13-4 are retrieved. After fusion they can either undergo two fates, one of which involves dissociation and return through the cytoplasm via non-vesicular transport. Alternatively they may diffuse out in the plasma membrane or remain trapped in the vicinity of the fusion site and be efficiently re-internalized. Fluorescence recovery after photobleaching experiments with munc13-4 and rab27a showed that rab27a is relatively stably associated with membranes (57) and predominantly in the GTP bond form (58). The turnover of munc13-4 on secretory lysosome membrane is also relatively slow and decreased ∼twofold after immune receptor signaling in rat basophil leukemia cells (59), suggesting that munc13-4 might be retrieved by coupling degranulation with endocytic re-uptake. In accord with this notion, Galandrini et al. recently found that munc13-4 was recycled back from PI(4,5)P2-enriched domains at the immunological synapse by AP-2 dependent endocytosis (33). The mechanism via which this occurs is remarkably similar to synaptic vesicle protein retrieval in presynaptic neurons. CD16 stimulation of human NK cells causes transient activation of Arf6 (60) which is essential for the recruitment of PIP5K-alpha and PIP5K-gamma to the plasma membrane, the formation of PI(4,5)P2 pools and secretion of secretory lysosomes (61). Upon fusion, granule membrane delivers munc13-4 to the PI(4,5)P2 pool generated by PIP5K-gamma (33). Presumably the association of munc13-4 depends on the C2B domain that has the ability to bind PI(4,5)P2 (62), which in turn serves as a recruitment hub for proteins regulating clathrin coated pit formation (63). Subsequent sorting processes in the endosomal system might then control delivery of re-internalized munc13-4 to its steady state localization in recycling endosomes (12, 13, 64).

## Posttranslational Modifications of Trafficking Proteins Regulating Degranulation

A central question is how the signals generated by cross-linking surface receptors with the corresponding ligands on target cells are transduced to the proteins that regulate transport and fusion of secretory lysosomes and how this modulates their function. SNAREs and their partners, and rabs play an essential role in degranulation, yet the mechanisms that determine the spatiotemporal control of their assembly in complexes for transport and fusion are incompletely understood. As protein kinases serve critical roles in reversible regulation of membrane transport (65), it is expected that phosphorylation of traffic proteins may modulate degranulation. In secretory lysosome exocytosis in mast cells, cross-linking of the high-affinity FcepsilonRI receptor leads to phosphorylation of SNAP-23 by IkappaB kinase 2, through a upstream PKC signaling pathway that is conserved in lymphocytes (66). Phosphorylation of SNAP-23 is essential for degranulation as ectopic expression of phospho-mimetic SNAP-23 mutant partially rescued the impaired IgE-mediated degranulation in IkappaB kinase 2-deficient mast cells (67, 68). Signaling cascades in platelets also cause phosphorylation of SNAP-23, which occurs on Ser95 and is a positive regulator of SNAP-23 dependent membrane fusion *in vitro* and platelet release *in vivo* (69). The gain in function of phosphorylated SNAP-23 correlates with its propensity to increase SNARE complex formation with syntaxin-11 and the R-SNARE VAMP8 that is critical for lytic granule exocytosis and cytotoxicity (36). Even though not yet shown in secretory lysosome release in immune cells, munc18-2 becomes phosphorylated during stimulatory conditions for regulated secretion in epithelial cells. As for SNAP-23, phosphorylation of munc18-2 enhances the assembly of a membrane fusion complex which serves as positive regulatory mechanism for fusion (70). Given the wide spread expression of munc18-2 (71), we anticipate that threonine/serine phosphorylation of munc18-2 and SNAP-23 has a general augmenting role in membrane fusion underlying secretory lysosome release (71).

## Perspective

The polarized secretion of lytic granule content in immune cells involves integration of major cytoarchitectural changes with precisely timed vesicular transport to the immunological synapse. Many proteins are known now that play a role in this traffic route and are important in degranulation. A major question is to understand how the signals that are initially generated upon encountering a target cell, control the proteins involved in membrane fission and fusion processes of this pathway. It is essentially unknown for instance if guanine nucleotide exchange factors (GEFs) and GTPase activating proteins (GAPs) for rab27a, and thereby the activity of the small GTPase are controlled upon signaling. This information could contribute insight in the order with which different rab27a effectors are recruited within a cell. We also need to improve our understanding where the proteins that control membrane traffic during degranulation are localized. Progress on this question will come from the application of powerful new imaging technologies that are increasingly being employed in immunological research. Ultimately such information will be crucial to annotate which step is controlled by which protein and how the network of traffic regulators cooperates in degranulation.

## Conflict of Interest Statement

The authors declare that the research was conducted in the absence of any commercial or financial relationships that could be construed as a potential conflict of interest.
